# Effect of MHC Haplotype on Immune Response upon Experimental SHIV_SF162P4cy_ Infection of Mauritian Cynomolgus Macaques

**DOI:** 10.1371/journal.pone.0093235

**Published:** 2014-04-02

**Authors:** Alessandra Borsetti, Flavia Ferrantelli, Maria T. Maggiorella, Leonardo Sernicola, Stefania Bellino, Alessandra Gallinaro, Stefania Farcomeni, Edward T. Mee, Nicola J. Rose, Aurelio Cafaro, Fausto Titti, Barbara Ensoli

**Affiliations:** 1 National AIDS Center, Istituto Superiore di Sanità, Rome, Italy; 2 Division of Virology, National Institute for Biological Standards and Control, Medicines and Healthcare products Regulatory Agency, South Mimms, Hertfordshire, United Kingdom; CEA, France

## Abstract

Little is known about the effects of Major Histocompatibility Complex (MHC) haplotypes on immunity to primate lentiviruses involving both acquired and innate immune responses. We present statistical evidence of the influence of MHC polymorphism on antiviral immunity of Mauritian cynomolgus macaques (MCM) following simian/human immunodeficiency virus SHIV_SF162P4cy_ infection, involving the production of pro- and anti-inflammatory cytokines and α-defensins, which may modulate acquired immune responses. During the acute phase of infection, IL-10 correlated positively with viral load and negatively with CD4+T cell counts. Furthermore, α-defensins production was directly correlated with plasma viral RNA, particularly at peak of viral load. When the effects of the MHC were analyzed, a significant association between lower anti-Env binding and neutralizing antibody levels with class IB M4 haplotype and with class IA, IB M4 haplotype, respectively, was observed in the post-acute phase. Lower antibody responses may have resulted into a poor control of infection thus explaining the previously reported lower CD4 T cell counts in these monkeys. Class II M3 haplotype displayed significantly lower acute and post-acute IL-10 levels. In addition, significantly lower levels of α-defensins were detected in class IA M3 haplotype monkeys than in non-M3 macaques, in the post-acute phase of infection. These data indicate that the MHC could contribute to the delicate balance of pro-inflammatory mechanisms, particularly with regard to the association between IL-10 and α-defensins in lentivirus infection. Our results show that host genetic background, virological and immunological parameters should be considered for the design and interpretation of HIV-1 vaccine efficacy studies.

## Introduction

Host genetic factors are important determinants that influence susceptibility to human immunodeficiency virus-1 (HIV)-1 infection and subsequent progression to acquired immunodeficiency syndrome (AIDS) [1], [2]. A set of genes are known to govern virus entry and/or development of effective innate and adaptive immune responses against the virus [3]. Among the immunogenetic determinants that are known to influence HIV/AIDS, MHC is involved in both innate and adaptive immunity and plays a primary role in the immune response [4], [5], [6], [7]. In the rhesus macaque simian immunodeficiency virus (SIV) model, disease progression to AIDS is clearly influenced by MHC class I and class II allelic polymorphism [8], [9], [10], [11], [12], [13], [14], [15]. In addition, pro- and anti-inflammatory cytokines, chemokines, and CD8+T cell anti-viral factors have been associated with complete or partial protection of naïve or vaccinated macaques against SIV/SHIV infection [16]. IL-10 is a pleiotropic cytokine that has immunomodulatory effects especially in down-regulating pro-inflammatory cytokines and costimulatory molecules, as well as major histocompatibility complex (MHC) class II proteins [17]. IL-10 has been associated with disease progression to AIDS but its role is still not clearly defined [18], [19], [20], [21]. In addition, recent studies have indicated that IL-15, which enhances innate and adaptive immunity by acting on CD8+T and natural killer cells, may play a role during acute HIV/SIV infection by impacting viremia and viral set point [16], [22], [23]. Also the human defensins HNP-1 to -3, which play an important role in innate immunity, have been reported to inhibit replication of R5 and X4 HIV-1 strains, including several primary isolates [24], [25], [26], [27], [28]. Therefore, both genetic and environmental factors may influence the susceptibility to infection.

Asian macaques have been extensively used for the preclinical evaluation of vaccine candidates, evidencing a different susceptibility to primate lentivirus-induced disease in different monkey species [29)]. In fact, the genetic diversity of the animals could contribute to determining the susceptibility of macaques to infection. This highlights the importance of considering host-related genetic background and immunological factors in the evaluation of vaccine efficacy in the different monkey species. In a recent study, we reported the effects of MHC class I and II haplotypes on CCR5-tropic SHIV_SF162P4cy_ infection in Mauritian cynomolgus macaques (MCM) [15].

To gain further insights into the genetic and immunological basis of natural resistance/susceptibility to infection, here we investigated the relationship between plasma cytokine levels, α-defensin levels, specific immunological (CD4+T cells, anti-Env binding and neutralizing antibodies) and virological (HIV DNA and RNA) parameters and MHC class I and II haplotypes in 21 MCM infected with SHIV_SF162P4cy_.

## Results

### Anti-Env antibody responses to SHIV_SF162P4cy_ infection

**Figure 1:**
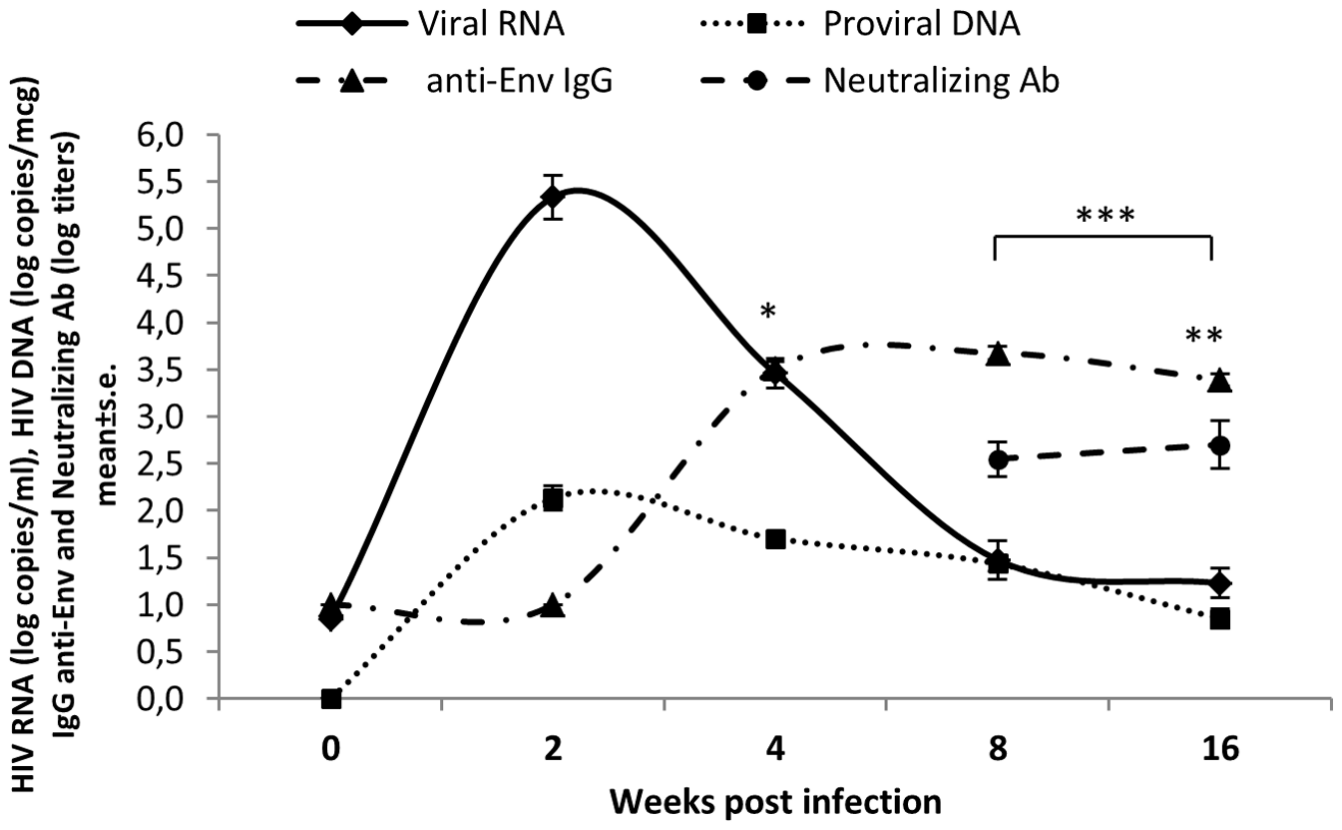
Dynamics of viral infection in 21 cynomolgus monkeys inoculated with SHIV_SF162P4cy_ during acute (2–4 weeks p.i.), post-acute (8–16 weeks p.i.) of infection. Data represent mean values with standard error of log plasma RNA load, log proviral DNA, IgG anti-Env Ab, and nAb from 0 to 16 weeks p.i. (*) week 4: anti-Env bAb titers correlated positively with viral load (p = 0.0002); (**) week 16: nAb titers correlated positively with anti-Env bAb titers (p = 0.0041); (***) post-acute phase: proviral DNA levels correlated positively with anti-Env bAb (p = 0.0225) and negatively with nAb titers (p = 0.0083).

Twenty-one MCM were challenged intrarectally with different doses of SHIV_SF162P4cy,_ a CCR5-tropic virus capable of establishing persistent infection and causing simian AIDS, similar to HIV disease in humans [15], [31], [32]. Whereas plasma viral load and proviral DNA were already evaluated in a previous work (15), here the dynamics of antibody responses was analyzed. (Fig.1). In infected macaques, plasma anti-Env bAb became detectable at week 4 p.i. and remained steady throughout the 16-weeks of follow-up, as well as homologous neutralizing antibody responses, measured 8 and 16 weeks p.i. (Fig.1). Anti-Env bAb titers correlated positively with viral load (p = 0.0002) and with nAb titers (p = 0.0041), at week 4 and 16, respectively (Fig. 1). When differences in post-acute infection were considered, proviral DNA levels correlated positively with anti-Env bAb (p = 0.0225) and negatively with neutralizing antibodies assessed against the challenge virus (p = 0.0083) (Fig.1).

### Plasma cytokine and α-defensin role in SHIV_SF162P4cy_ infection

**Figure 2:**
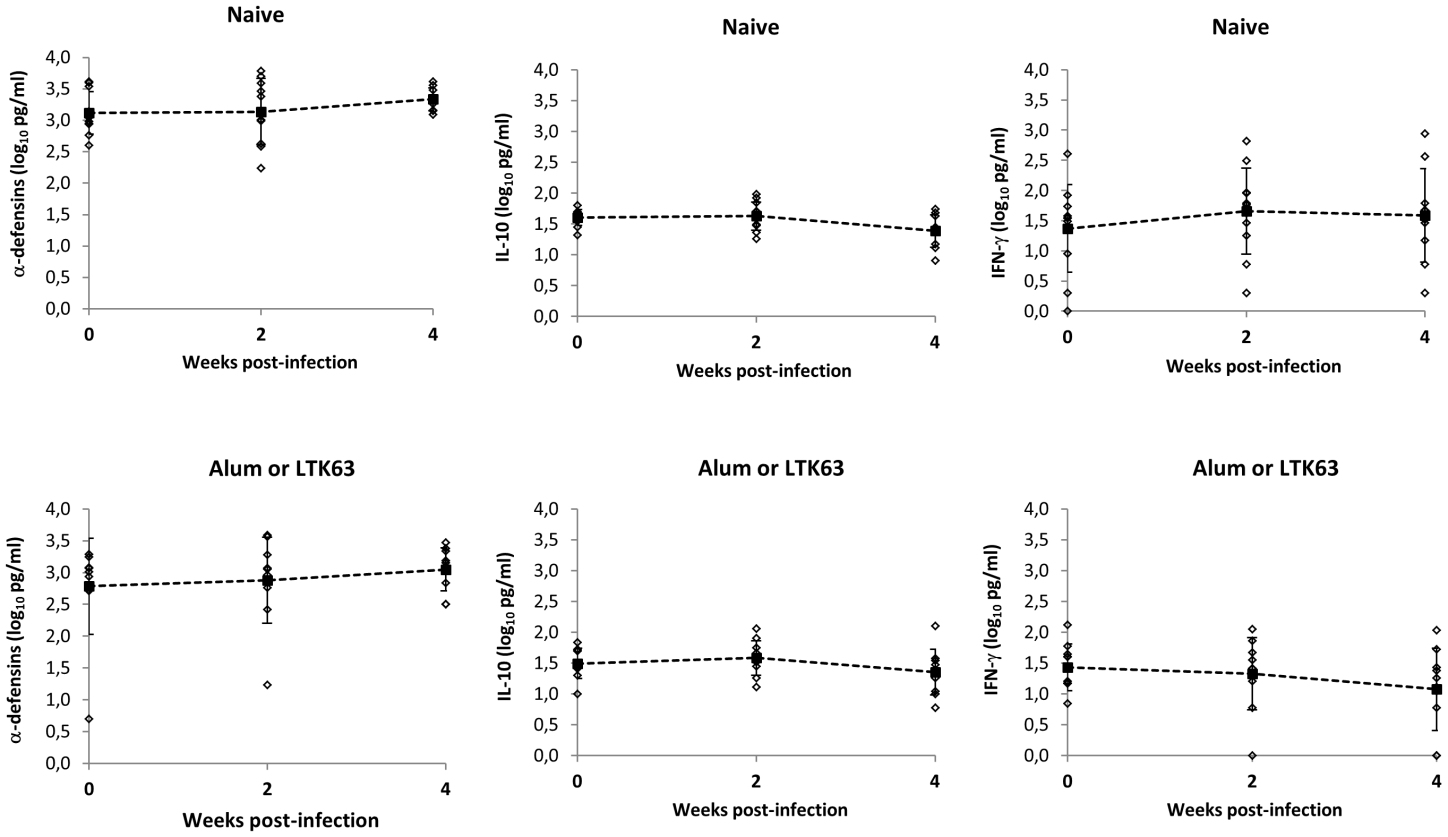
Plasma α-defensin, IL-10 and IFN-γ levels with respect to the pre-infection values in naïve (n = 11) and adjuvant treated monkeys (n = 10) inoculated with SHIV_SF162P4cy_. No significant differences were observed between naive and adjuvant treated monkeys during the observation period. Mean values with standard deviations are connected by dotted lines.

In order to evaluate the role of antiviral innate immunity following SHIV_SF162P4cy_ infection, the production of cytokines (IL-10, IFNγ, IL-15) and α-defensins were determined in plasma of infected monkeys. To exclude a bias in the results due to prior Alum or LTK adjuvant treatments of some animals, analysis of viral load, proviral load and cytokine and α-defensin production of all monkeys were assessed during acute and post-acute phases of infection. No significant differences were observed between adjuvant treated monkeys and untreated animals. Plasma levels of α-defensins, IL-10 and IFNγ peaked in the acute phase of infection (Fig.2), whereas IL-15 showed fluctuation throughout the study (data not shown). To assess the relationship between cytokine production and SHIV-1 replication in the acute phase of infection, longitudinal regression model was applied to viral load, including α-defensins, IFNγ, IL-10 and IL-15 as explicative factors.

**Figure 3:**
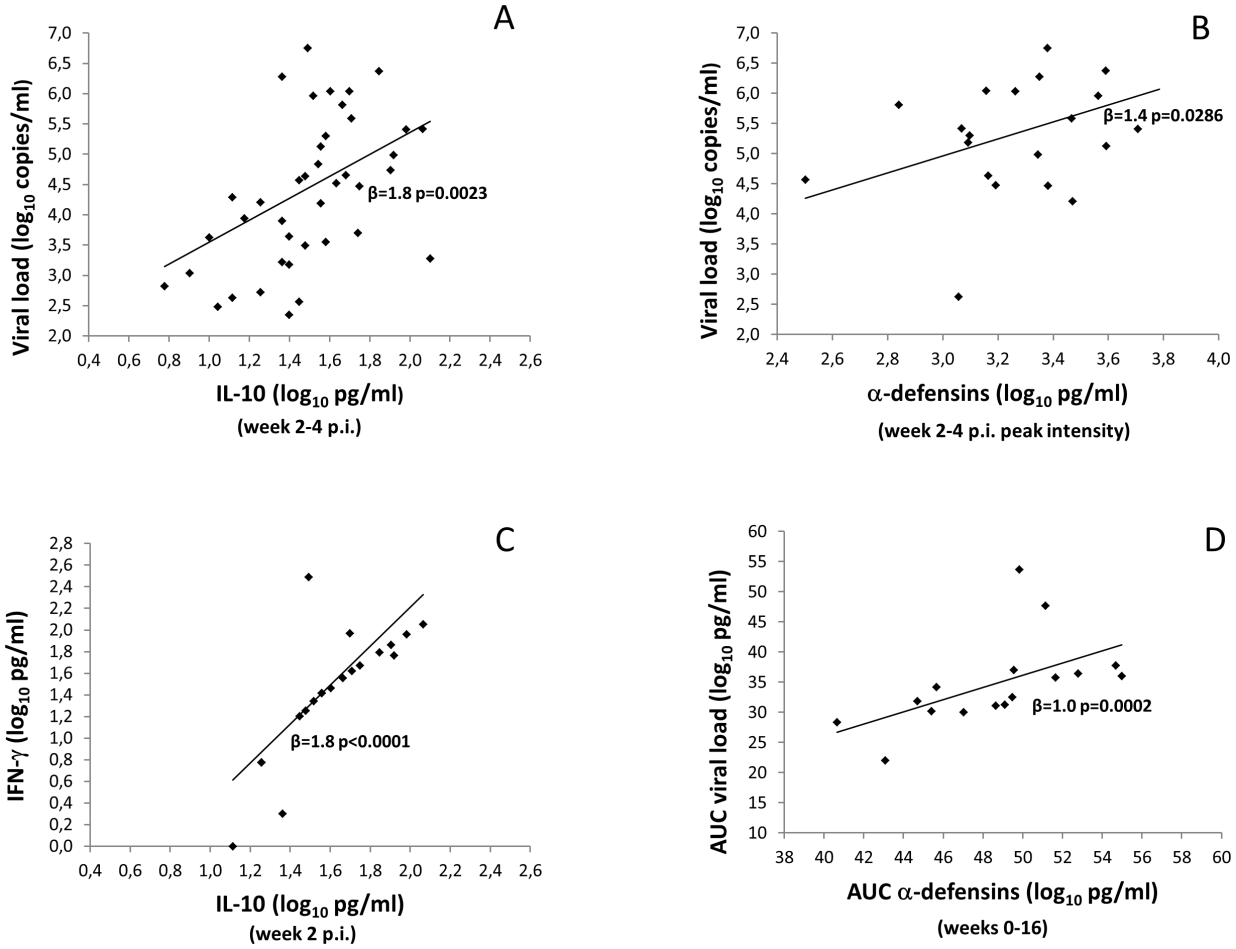
Regression analyses between immunological and virological parameters in infected monkeys during the acute phase of SHIV_SF162P4cy_ infection. A significant relationship was detected between: A) IL-10 production and plasma viral load (p = 0.0023); B) α-defensins production and viral RNA (p = 0.0286); C) IFNγ and IL-10 production (p<0.0001). D) AUC: significant relationship between viral load and α-defensins (p = 0.0002).

The analyses showed that during the acute phase of infection IL-10 production was related to plasma viral load (p = 0.0023) (Fig. 3A), and a significant relationship was also detected between α-defensins and viral RNA at the peak of viral load (p = 0.0286) (Fig. 3B). Moreover at week 2 p.i., higher levels of α-defensins were observed in viremic animals as compared to exposed but aviremic animals (1084 pg/ml, range 17–6093, vs 439 pg/ml, range 5–1317, p = 0.0276 Wilcoxon rank-sum test). Despite neutrophils are the major cellular source of α-defensins *in vivo* [24], no significant correlation was observed between plasma levels of α-defensins and neutrophils counts in viremic or aviremic monkeys during the study (data not shown).

Previous reports have indicated that plasma IL-10 protein levels are elevated during acute HIV infection [21], [33]. As IL-10 is a major inhibitory immunoregulator, we next examined whether IL-10 levels were associated with distinct cytokine profiles in peripheral blood. Interestingly, a significant relationship between IFNγ and IL-10 was detected during the acute phase of infection and in particular at week 2 (p<0.0001) (Fig. 3C), whereas no significant correlation was detected between α-defensin and IL-10 during the acute or post-acute phase of infection. When the area under the curve (AUC) for virological and immunological parameters was analyzed, it was found a significant relationship between viral load and α-defensins from 0 to 16 weeks p.i. (p = 0.0002) (Fig.3D). Lastly, no correlation was found between IFNγ or IL-15 and plasma viremia, proviral DNA and CD4^+^ T or CD8^+^ T cell counts.

### Effects of MHC haplotypes on anti-Env binding and neutralizing antibodies

**Figure 4:**
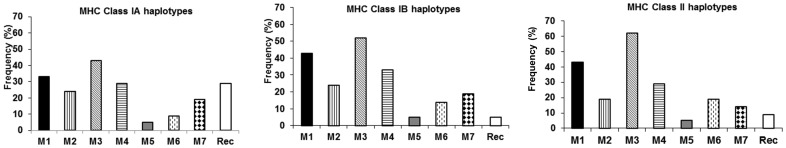
Frequency of MHC class IA, class IB and class II and recombinant haplotypes in animals included in the study. Animals carrying specific Mhc class I haplotypes. MHC class IA: M1 = 7, M2 = 5, M3 = 9, M4 = 6, M5 = 1, M6 = 2, M7 = 4, rec = 6; frequency of MHC class IB: M1 = 9, M2 = 5, M3 = 11, M4 = 7, M5 = 1, M6 = 3, M7 = 4, rec = 1; frequency of MHC class II: M1 = 9, M2 = 4, M3 = 13, M4 = 6, M5 = 1, M6 = 4, M7 = 3, rec = 2 *rec* recombinant haplotype.

In a previous study, MHC class I and II haplotypes in SHIV_SF162P4cy_-infected monkeys were defined by microsatellite analysis, with the alleles for class I and class II regions being inferred on the basis of established haplotype allele associations. Seven haplotypes (M1 to M7), or simple recombinants thereof, were detected. M3 was the most commonly represented haplotype in the cohort, whereas M5 was found only in one animal (Fig. 4). Our recent data indicated that M4 haplotype impacts on proviral copy number and CD4+T cell counts. In particular, lower proviral DNA values in MHC class IA M4 macaques and lower CD4+T cell counts in MHC class IA, IB, and class II M4 animals were detected during the acute and chronic (24–46 weeks) phases of infection, respectively [15]. In the present study, the influence of MHC haplotype on humoral response was analyzed.

**Figure 5:**
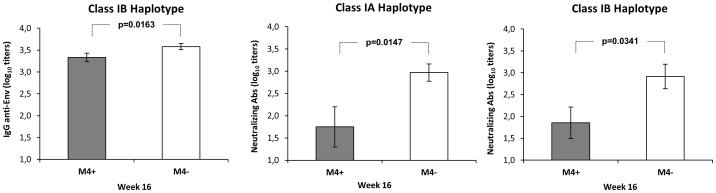
Effects of MHC class I*A* and I*B* haplotypes on IgG anti-Env antibodies and neutralizing Ab following infection with SHIV_SF162P4cy_. Mean values with standard error are depicted for all animals positive or negative for the indicated haplotype: M4 (n = 6) non-M4 (n = 15) class I*A*; M4 (n = 7), non-M4 (n = 14) class I*B*.

At week 16 p.i., lower anti-Env bAb were associated with the MHC class IB M4 haplotype (p = 0.0163) (Fig.5A) and lower nAb were found in MHC class IA and IB M4 animals (p = 0.0147 and p = 0.0341) (Fig.5B). We therefore speculated that during the early phase of infection M4 animals were poorly able to control infection as a consequence of a limited antibody response. This would explain the lower CD4 T cell counts and the resultant reduction in virus target cells leading to lower proviral loads.

### Lower IL-10 and α-defensin plasma levels in MHC haplotype M3 infected monkeys

**Figure 6:**
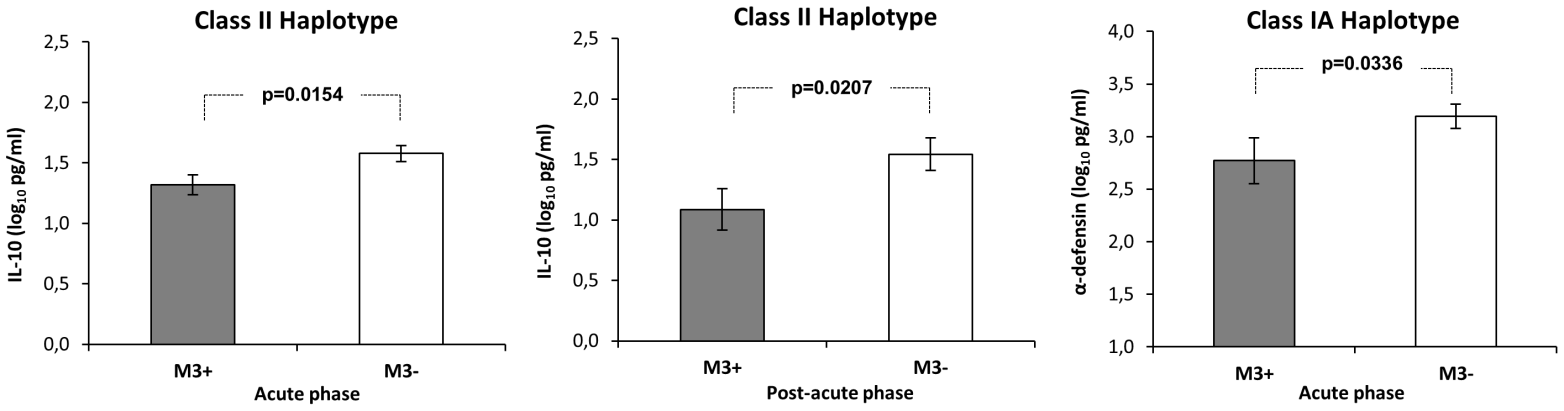
Effects of MHC class I*A* and II haplotypes on IL-10 and α-defensin production during SHIV_SF162P4cy_ infection. Mean values with standard error are depicted for all animals positive or negative for the indicated haplotype: M3 (n = 9) non-M3 (n = 12) class I*A*; M3 (n = 13) non-M3 (n = 8) class II.

We investigated whether the MHC haplotype could influence the production of IL-10 and α-defensins in the infected monkeys. We found significantly lower levels of IL-10 during the acute (*p* = 0.0154) and post-acute (*p* = 0.0207) phases of infection associated with the MHC class II M3 haplotype (n = 13) (Fig.6A). Next, we found that the M3 haplotype also impacted on α-defensin production. In particular, MHC class IA M3 monkeys had lower levels of α-defensins than non-M3 macaques in the acute phase of infection (*p* = 0.0336) (Fig.6B).

## Discussion

We report here the analysis of specific Ab responses, including nAb, plasma cytokines and α-defensins in the context of clinical and virological parameters, and MHC genetics in cynomolgous macaques infected with SHIV_SF162P4cy_.

The temporal relationship between appearance of a virus-specific immune response, reduction of viremia, and resolution of the acute viral syndrome has been clearly established ([34], [35], [36]. In fact, early HIV/SIV-specific antibodies, either alone or in conjunction with other components of the immune system, may contribute to the control of virus replication [37]. In particular, understanding the relationship between binding and neutralizing antibody titers is important to characterize factors in HIV-1 pathogenesis and disease progression that could be indicators or correlates of protection for vaccine development. Our study shows that although levels of anti-Env binding antibodies paralleled those of neutralizing antibodies at week 16 p.i., only neutralizing antibodies appeared to correlate with a control of proviral DNA in the post-acute phase.

The levels of other immunological markers, also changed over the course of infection. After challenge, IL-10 and IFN-*γ* levels were higher in the infected monkeys, with differences being statistically significant for IL-10. The direct effects of IL-10 on virus production are complex. In humans, lack of a clear correlation of IL-10 plasma levels with viral load in specific phases of HIV-1 infection as well as IL-10 fluctuations observed even in healthy subjects make it difficult to rely on plasma IL-10 as a biomarker for HIV-1 pathogenesis. In fact, even though IL-10 is elevated in plasma during chronic HIV-1 infection, with levels that correlate with HIV-1 viral load [21], higher levels of IL-10 expression were also associated with significantly higher plasma viral loads in acute infection [33].

Our data in macaques confirm the latter results obtained in humans, demonstrating a direct correlation between elevated plasma IL-10 levels and viral load during acute infection, concurrent with very high HIV viral load, which declined after the resolution of primary infection [33]. Moreover IL-10 levels correlated directly with HIV-1 viral load and inversely with CD4 counts, which is also consistent with previous evidence in humans [38]. Overall, the significant positive correlation of IFN-*γ* with IL-10 expression suggests that, as the production of pro-inflammatory cytokines increases, the production of IL-10 also increases to reduce inflammation and activation. Given the crucial role of immune activation in HIV disease progression, it is possible that despite its inhibitory effect on T cells and the mRNA upregulation in multiple cell types of HIV-infected subjects, IL-10 has a net beneficial impact during later stages of HIV infection by limiting systemic immune hyperactivation and CD4 T-cell loss.

At the peak of viral replication, viremic animals showed a statistically significant increase in *α*-defensins levels, which decreased in parallel with viral load in the post-acute phase. This trend was not observed in exposed but aviremic macaques. In line with this result, at week two p.i. levels of α-defensins were significantly higher in viremic as compared to aviremic animals. It is conceivable that the increase in α-defensins production and release by cells implicated in the early innate immune response during acute infection was strictly related to plasma viremia, even though no significant correlation was detected between α-defensin and IL-10 during acute and post-acute phases of infection. It could be possible that in the viremic animals belonging to this study, IL-10 influences indirectly α-defensin release, as a consequence of the attenuation of neutrophil extracellular traps (NETs) production, which results into a significantly reduced virucidal capacity of neutrophils [39], [40]. This mechanism has recently been reported by Saitoh et al. [40]. NETs capture HIV-1 by TLR7 and TLR8 and promote HIV-1 elimination through myeloperoxidase and α-defensin. HIV-1 counteracts this response by inducing CD209-dependent production of IL-10 in dendritic cells that modulates neutrophil function and attenuates NET release. Although these studies were carried out using an in vitro cell culture system, authors speculate that NET formation could occur in the mucosa as well as in the blood [40]. It will be important to understand how these mechanisms function within the context of the host immune response to viruses in vivo. Also MHC types were shown to significantly influence IL-10 production [21]. We observed that the lower IL-10 levels assessed during the acute and post-acute phases of infection in MHC class II M3 haplotype animals were concomitant with the lower α-defensin values associated with MHC class IA M3 in acute infection. To explain this result, it should be considered that high concentrations of α-defensins are present in the vaginal epithelium as well as in Paneth cells of the small intestine [41]. Inflammation causes the infiltration of neutrophils across intestinal epithelia in response to pro-inflammatory cytokines and β-chemokines, which release large amounts of α-defensins. It is possible that α-defensins exert anti-HIV activity at sites of active inflammation, mainly at the mucosal level such as in the gut-associated lymphoid tissue, and have immunomodulatory effects including an increased production of pro-inflammatory cytokines and probably explaining the higher levels of α-defensins recovered in plasma of animals inoculated by intrarectal route. At the same sites, HIV-1 could induce CD209-dependent production of IL-10 by dendritic cells to suppress NET formation. Since virus RNA levels and cytokine levels in plasma may differ in the mucosa as compared with the blood [42], in order to formally investigate this hypothesis, one should examine IL-10 and α-defensin expression levels in regional and systemic lymphoid tissue as well as in gut-associated lymphoid tissue in M3 haplotype macaques as compared to the other haplotypes. It is possible that the lower levels of α-defensins displayed early in infection by the MHC class IA M3 resulted from the Mafa-A2*05∶11 allele and relative transcripts, which are usually low [43]. In fact, M1, M2, and M3 class IA haplotypes transcribe an identical Mafa-A4*01∶01 allele and highly similar Mafa-A1*063 alleles, while only the M3 haplotype encodes the allelic variant Mafa-A2*05∶11. However, since these animals are M3 for half of their MHC genes, both MHC haplotypes should be considered. As already suggested by other authors [44], the possibility that genes on the non-M3 haplotype modulate the effect of the M3 haplotype, or vice versa, cannot be excluded. To strengthen the results obtained from the M3 class I*A* haplotype animals, one could study homozygous animals for the same MHC allele and expand the case study including a larger number of animals.

The role that MHC haplotypes play in antibody response in the control of virus infection are uncertain [35], [44]. In a previous work we found that the lower CD4+T cell levels observed in class I*A*, I*B* and II M4 haplotype animals in the chronic phase of infection were concomitant with the lower proviral DNA values associated with class I*B* and II M4 haplotype in acute infection [15]. Here we observed that at week 16 class IB M4 and class IA and IB M4 were associated with lower anti-Env binding and neutralizing antibodies, respectively. These results support the hypothesis that early in infection M4 animals have a weak antibody control over the virus, hence the marked drop in CD4+T cells and therefore a reduction in virus target cells leading to lower proviral loads. Unfortunately, cellular immunity to virus proteins that may also contribute to explain the outcome of infection in animals could not be determined.

Presuming that early events during acute infection may affect the capacity of a vaccine to block acquisition of infection, different virological and immunological parameters of acute SIV/SHIV infection should be considered for the design and interpretation of HIV-1 vaccine efficacy studies.

## Conclusions

In summary, we have presented for the first time evidence of the differential control of immune response by both MHC class I and II polymorphism on the replication of a cynomolgus macaque-derived R5-tropic SHIV by comparing the combination of virological and immunological parameters of infection. We conclude that MHC polymorphism can influence antiviral immunity of MCM during infection, modulating the delicate balance of immunomodulatory factors, such as IL-10 and α-defensins, and anti-Env binding and neutralizing antibody responses.

## Material and Methods

### Ethics Statement

Twenty-one adult male cynomolgus macaques (*Macaca fascicularis*) were imported, with CITES permit, from the Mauritius breeding colony (Mauritian cynomolgus macaques, MCM). Animals were matched for age and weight. All monkeys were negative for infections with simian immunodeficiency virus (SIV), type D simian 1, 2, 3, 5 retroviruses (SRV) and simian T cell leukaemia (STLV-I) viruses, simian herpes B virus, cytomegalovirus, Ebola and Marburg viruses. Animals were housed at the National AIDS Center, Istituto Superiore di Sanità (ISS), in single cages in an authorized P3 facility in single stainless steel cages according to the National (Ministry of Health D.L. 27/1/1992 n. 116) and European guidelines for non-human primate care (ECC, Directive No. 86–609, Nov. 24, 1986; Refinement, Reduction and Replacement towards the use of animals for scientific procedures. 99/167/EC: Council Decision of 25/1/99) under the full care of veterinarians. All procedures were performed to minimize suffering, improve housing conditions, and to provide enrichment opportunities (e.g., varied food supplements, foraging and task-oriented feeding methods, interaction with caregivers and research staff, view of the other monkeys). The temperature was maintained at 21–23 uC and humidity ranged from 50 to 60% with 10–15% air changes per h. The light cycle was 12-h light/12-h dark. Animals were fed a commercial maintenance chow (Rieper, Mucedola s.r.l., Settimo Milanese, Italy) supplemented biweekly with fresh fruit. Water was supplied ad libitum. Animal experiments were approved by the Quality and Safety Committee for Animal Trials of the ISS. All clinical procedures were performed upon anesthesia with 7–10 mg/kg Zoletil (a combination of Tiletamine and Zolazepam). Animals were examined clinically and weight and rectal temperature were measured while under ketamine hydrochloride anaesthesia (10 mg Kg -1, intramuscularly). Blood samples for haematological analysis and for immunological and virological assays were taken in the morning prior to feeding. All monkeys were observed daily for behavior, local reactions after immunizations, and clinical signs of disease. Serum biochemical/hematological parameters and animal weight were assessed on a regular basis, according to the blood drawing schedule. No local reactions were recorded after immunizations and all safety-related parameters evaluated were in the normal range at all times. All animals lived throughout the study. The animal protocols (115/2005-C, 75/2006-B, 169/2008-C, 170/2008-C) were approved by the ethics committee of the Istituto Superiore di Sanità. All clinical procedures were performed upon anesthesia with 7–10 mg/kg Zoletil (a combination of Tiletamine and Zolazepam).

### Animals

Macaques used in this study were part of different experimental protocols as untreated or adjuvant-treated control animals. The latter were treated with different adjuvants such as aluminium phosphate (Alum), 250 μl, administered subcutaneously (s.c.) or nontoxic mutant of the heat-labile enterotoxin (LTK63), 30 μl, administered intranasally (i.n.). LTK63 is an *Escherichia coli* heat-labile enterotoxin mutant, which proved generally safe and effective as an intranasal adjuvant both in animals and in humans [30]. Monkeys AH694, AK407, AK484, AK803, AK952, and AL639 received LTK63 at weeks 0, 4, 8 and Alum at weeks 24 and 36 before virus challenge performed at week 44; monkeys AQ271, AP511, and AQ882 were administered with Alum at weeks 23 and 36 before challenge performed at week 48; monkey AF318 received Alum at weeks 0, 4, 12, 23, 43, 87, and LTK63 at weeks 57 and 67 before challenge performed at week 96. The challenge virus was the SHIV_SF162P4cy_, obtained as previously described [15], [31] and titrated *in vivo* in cynomolgous macaques. Animals were inoculated intrarectally with different doses, ranging from 1.79 to 179 MID_50_, of the same SHIV_SF162P4cy_ virus stock. The 21 monkeys included in this study were overtly infected, with detectable plasma viremia and cell-associated SHIV DNA at multiple time points (Fig.1A). Besides these animals, a reference group of 6 aviremic male cynomolgous macaques was also considered in some of the analyses performed, as indicated in the Results. These animals were challenged intrarectally with a low dose (range: 0.0179–1.79 MID_50_) of the same virus stock as the viremic macaques, yet resulted always negative for viral RNA, proviral DNA and anti-Env antibodies. Nonetheless, they showed a mild transient CD4+T cell count decline at week 2 post infection (p.i.) (data not shown).

### Plasma viral RNA and proviral DNA detection

Plasma levels of SHIV_SF162P4cy_ were determined using a “one step” real time RT-PCR (RNA-polymerase chain reaction assay), with a threshold limit for detection of 50 RNA eq/ml [32]. To quantitate the cell-associated viral load, DNA was extracted from 400 μl of whole citrated blood by using the QIAmp DNA Blood Mini Kit (QIAGEN, Milan, Italy) according to the manufacturer's instructions. SHIV proviral copies were determined using Taqman real-time PCR as previously described [32]. The lower limit of detection of this assay was 1 SHIV proviral copy/μg of DNA.

### Cytokine, chemokine and α-defensin detection by ELISA

Monkey IL-10, IFNγ (Cell Sciences, Canton, MA, USA), α-defensins (Hbt Human HNP 1–3, Hycult Biotechnology, Uden, The Netherlands) and IL-15 (human IL-15 from R&D Systems) concentrations in plasma of healthy and experimentally infected cynomolgus monkeys were determined with commercial high-sensitivity enzyme immunoassay ELISA kits according to the manufacturers' instructions (detection range for IL-10 and IFNγ: 5–320 pg/ml; for α-defensins: 41–10,000 pg/ml; for IL-15: 3.9–250 pg/ml). Each sample was tested in duplicate. Samples from a given animal were analyzed in the same microtiter plate to minimize run-to-run variability. The intra- and inter-assay coefficients of variation were <10% for all ELISAs. Levels of IL-10, IFNγ, IL-15, and α-defensins were also measured in healthy controls housed at Istituto Superiore di Sanità (ISS) to determine the physiological fluctuations over time.

### Microsatellite analysis and allele-specific PCR

MHC class I*A* and I*B* and class II haplotypes were determined by microsatellite PCR with resolution of recombinant class I*B* haplotypes by allele-specific PCR as previously described [12].

### Detection of anti-Env binding antibodies

For the assessment of Env-specific IgG, ELISA plates were coated with an oligomeric, SF162 strain-derived, V2 loop-deleted Env (EnvΔV2) protein and the assay performed as reported [31]. Binding Ab (bAb) titers were calculated as the reciprocal plasma dilution giving optical density (OD) readings >3 standard deviations (SD) above negative control, normal MCM plasma samples [31].

### Neutralization assay

Plasma neutralizing antibodies (nAb) were assessed using a viral infectivity assay based on TZM-bl cells infected with SHIV_SF162P4cy_ [31]. Percent neutralization was calculated relative to the negative control infection, containing pre-challenge plasma of the same monkey. nAb titers were estimated as the reciprocal plasma dilution resulting in 50% inhibition of infection (ID_50_).

### Statistical methods

All parameters included in the analyses were evaluated for different phases of infection: acute (2–4 weeks) and post-acute (8–16 weeks). Log10 transformation was performed to normalize the data distribution of viral load, proviral DNA, anti-Env IgG, bAb titers, nAb titers and cytokines. Relationship among virological and immunological parameters was assessed by the regression model or by the longitudinal analysis using the generalized estimating equations method (GEE). The trapezium method was used to estimate the area under the curve (AUC) for the whole follow-up period. The effects of MHC haplotypes on humoral responses and on cytokines plasma concentration were analyzed by the longitudinal analysis of variance by GEE method, where all factors were adjusted for each other. Statistical analyses were carried out at two-sided with a 0.05 significance level, using SAS software, version 9.2 (SAS Institute, Cary, NC).
